# Milk-derived miRNA profiles elucidate molecular pathways that underlie breast dysfunction in women with common genetic variants in *SLC30A2*

**DOI:** 10.1038/s41598-019-48987-4

**Published:** 2019-09-03

**Authors:** Shannon L. Kelleher, Annie Gagnon, Olivia C. Rivera, Steven D. Hicks, Molly C. Carney, Samina Alam

**Affiliations:** 1 Department of Biomedical and Nutritional Sciences, University of Massachusetts Lowell, Lowell, Massachusetts United States of America; 2 Department of Cellular and Molecular Physiology, Penn State Hershey College of Medicine, Hershey, Pennsylvania United States of America; 3 Department of Surgery, Penn State Hershey College of Medicine, Hershey, Pennsylvania United States of America; 4 Department of Pediatrics, Penn State Hershey College of Medicine, Hershey, Pennsylvania United States of America

**Keywords:** Reproductive biology, Predictive markers

## Abstract

Studies in humans and pre-clinical animal models show milk-derived miRNAs reflect mammary gland function during lactation. The zinc transporter *SLC30A2*/ZnT2 plays a critical role in mammary gland function; ZnT2-null mice have profound defects in mammary epithelial cell (MEC) polarity and secretion, resulting in sub-optimal lactation. Non-synonymous genetic variation in *SLC30A2* is common in humans, and several common ZnT2 variants are associated with changes in milk components that suggest breast dysfunction in women. To identify novel mechanisms through which dysfunction might occur, milk-derived miRNA profiles were characterized in women harboring three common genetic variants in *SLC30A2* (D^103^E, T^288^S, and Exon 7). Expression of ten miRNAs differed between genotypes, and contributed to distinct spatial separation. Studies in breast milk and cultured MECs confirmed expression of ZnT2 variants alters abundance of protein levels of several predicted mRNA targets critical for breast function (PRLR, VAMP7, and SOX4). Moreover, bioinformatic analysis identified two novel gene networks that may underlie normal MEC function. Thus, we propose that genetic variation in genes critical for normal breast function such as *SLC30A2* has important implications for lactation performance in women, and that milk-derived miRNAs can be used to identify novel mechanisms and for diagnostic potential.

## Introduction

MicroRNAs (miRNAs) are small, non-coding nucleic acid sequences (~22 bases) that regulate post-transcriptional gene expression by binding to a specific mRNA target and either inhibiting translation or promoting degradation^1^. One miRNA can target over 100 genes, and one gene may be regulated by multiple miRNAs^2^. In mammalian cells, miRNAs are predicted to regulate over 60% of protein synthesis (reviewed by Cui *et al*.)^3^, and the interactions between miRNAs and protein-coding genes appear to comprise genetic interactomes^4^, permitting global regulation of numerous processes. In addition to controlling normal physiology, miRNAs have been implicated in various pathological states such as cancer, autoimmune disease, gastrointestinal disease, and reproductive system disorders.

High levels of miRNAs are detected in secreted body fluids including serum, urine, saliva, seminal fluid, and milk^3^. Milk is one of the richest sources of miRNAs in humans and contains ~1400 mature miRNAs, with the bulk of miRNAs in human milk being enriched in the lipid and cellular fractions^5^. Milk-derived miRNAs are also secreted by the lactating mammary epithelial cell (MEC) in exosomes^6^, which are microvesicles of ~30–100 nm that are packaged and secreted into extracellular fluids. Recently, there has been much interest in milk-derived miRNAs as potential regulators of the neonatal gastrointestinal and immune systems^7^. A recent report by Alsaweed and colleagues^6^ suggests the profile of milk-derived miRNAs may also reflect lactation performance and breast health. Consistent with this idea, miRNA profiles of mammary gland tissue distinguish discrete stages of mammary gland development in mice, paralleling profound increases in the expression of genes required for ion transport, G protein signaling, translation and intracellular protein transport, and decreases in inflammatory response, cell division, Wnt signaling, carbohydrate metabolism and oxidative phosphorylation^8^. Studies in dairy cows show miRNA profiles in mammary gland tissue differ dramatically between lactating and non-lactating periods; ~11% of miRNAs are differentially expressed between these physiological states, and ~2% were only expressed during lactation^9^. Moreover, studies in rats show miRNA profiles in mammary gland tissue change throughout lactation, reflecting changes in the functionality of the mammary gland as lactation and involution proceed^10^. Few studies characterizing differences in milk-derived miRNAs in women have been conducted; however, we recently reported the profile of milk-derived miRNAs in preterm milk is profoundly different from that in milk from women who gave birth at term^11^. We speculate this reflects the immaturity and sub-optimal performance of the preterm mammary gland, and suggests milk-derived miRNAs reflect the functional capacity of the mammary gland during lactation, and thus may serve as bioreporters of lactation performance.

**Figure 1:**
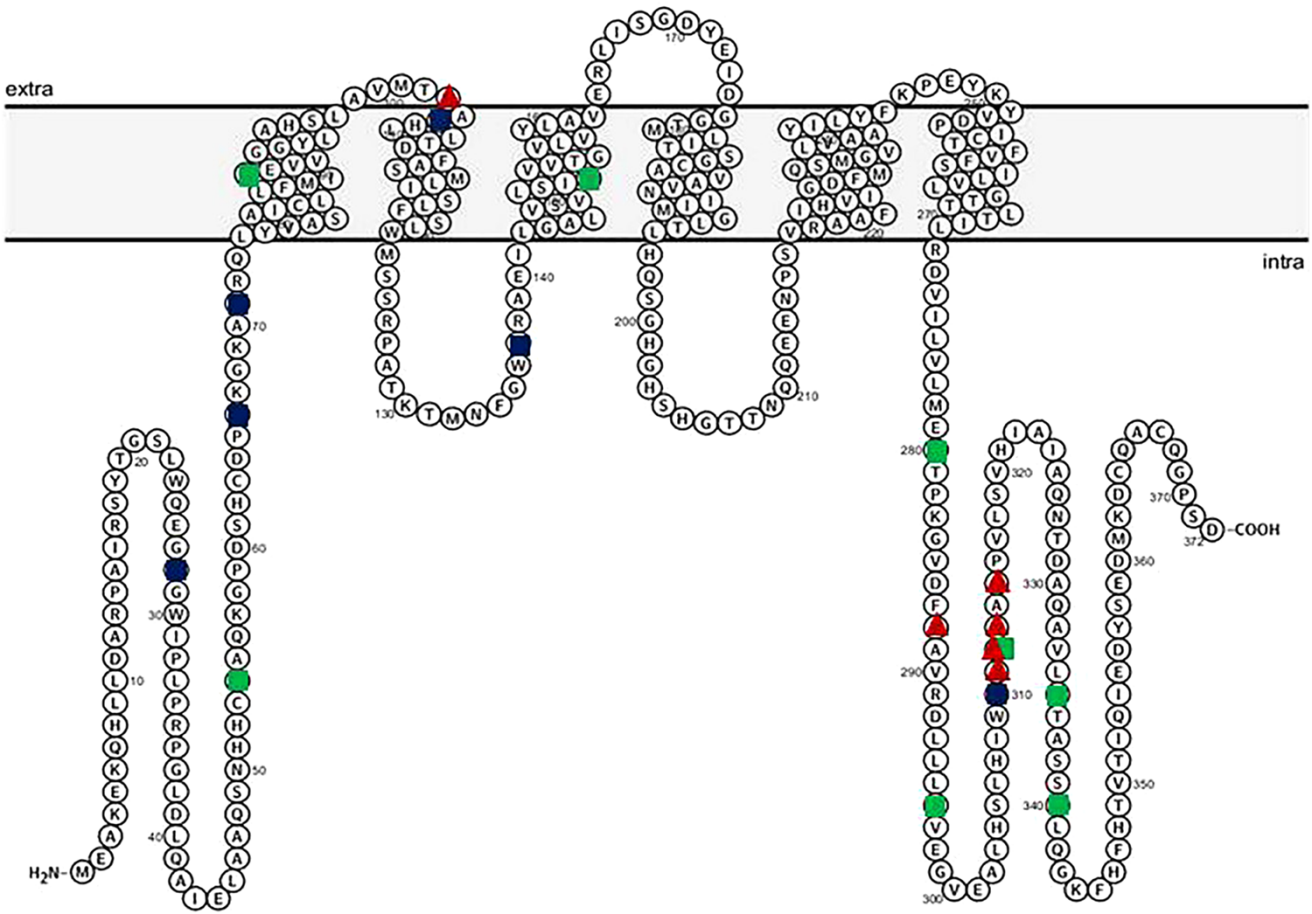
Diagram of the predicted secondary structure of ZnT2. Protter v 1.0 (http://wlab.ethz.ch/protter/#) was used to generate the predicted secondary structure of ZnT2. Mutations (green circles) and non-synonymous variants (blue circles) we previously identified in breastfeeding women are noted. Common non-synonymous variants explored in this report (D^103^E, T^288^S and Exon 7) are identified by red triangles.

The zinc transporter ZnT2 (*SLC30A2*) is highly expressed in MECs^12^. ZnT2 has six transmembrane domains and functions as a dimer^13^ to import zinc into mitochondria^14^ and vesicles^12^. In addition to playing a critical role in zinc secretion into milk, ZnT2 is critical for MEC differentiation and polarity, milk secretion and composition^15^. Moreover, studies in mice and cultured MECs show ZnT2 is redistributed from secretory vesicles to lysosomes in response to tumor necrosis factor α (TNFα), promoting lysosomal biogenesis and cell death, which are critical during early involution^16,17^. In humans, seven missense mutations have thus far been identified in *SLC30A2* that are associated with a 50–90% reduction in milk zinc concentration and result in severe neonatal zinc deficiency in exclusively breastfed infants^18–21^. However, ZnT2 variants are likely quite common, as Golan and colleagues recently estimated the frequency of loss-of-function mutations to be 1 in 2334^22^. Indeed, we recently reported that non-synonymous ZnT2 variants are quite common in humans; 36% of a random population of breastfeeding women harbored non-synonymous ZnT2 variants. Moreover, most of these variants were functionally compromised and lead to either loss- or gain-of-function, and consequent changes in cellular zinc management, alterations in cell cycle, and cell death *in vitro*^23^. Three of these ZnT2 variants were particularly common (D^103^E, 9%; T^288^S, 16%; and a series of compound substitutions located in exon 7 consisting of T^288^S, L^311^V, T^312^K, V^313^G, Q^315^R and referred to herein as “Exon 7”, 7%)^23^. The Exon 7 variant lies within the C-terminus and is predicted to be in a cytoplasmic domain (Fig. 1)^24^. While women who harbor the Exon 7 variant have milk zinc levels ~twice as high as women who harbor two wild-type alleles^23^, the underlying cellular pathology of Exon 7 has not yet been elucidated. The D^103^E substitution is predicted to be in the first intraluminal domain (Fig. 1). Women who harbor D^103^E have ~30% less zinc in their milk compared to women who harbor two wild-type alleles^23^. Functional studies *in vitro* suggest that D^103^E is retained in the endoplasmic reticulum (ER) and lysosomes, and results in “loss-of-function” and significant shifts in cell cycle^23^. While this is consistent with the observation that women who harbor D^103^E have modestly elevated milk sodium levels, a classic hallmark of breast dysfunction^25–28^, the molecular defects are not yet understood. The T^288^S substitution is also predicted to be located within the cytoplasmic C-terminus (Fig. 1). Women who harbor T^288^S also have ~30% less zinc in their milk, concurrent with significantly elevated milk sodium levels^23^. Further characterization of T^288^S recently showed their milk also contains molecules indicative of increased oxidative and ER stress, and mammary gland remodeling^29^. Studies *in vitro* showed T^288^S is retained in the ER and lysosomes leading to zinc accumulation, ER and oxidative stress, and the activation of STAT3 signaling, a hallmark of mammary gland remodeling during involution^29^. This provides compelling evidence that expression of genetic variants in ZnT2 may compromise breast function; however, the molecular pathways that underpin these consequences are not understood. Here we conducted a pilot study and used milk-derived miRNA profiling to identify molecular pathways affected in women harboring common non-synonymous genetic variants in ZnT2 in hopes of providing insight into the consequences on MEC function and lactation competence.

## Results

### Patterns of miRNA expression

**Figure 2:**
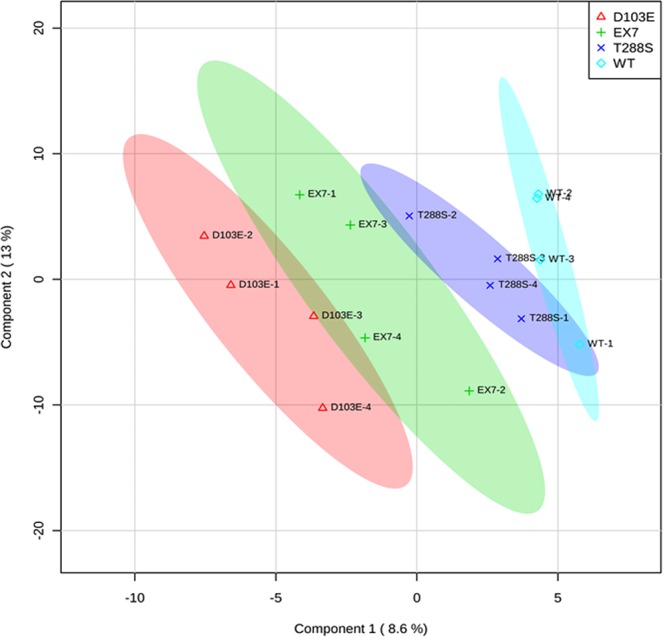
Partial least squares discriminant analysis achieves spatial separation of milk-derived miRNA profiles. A two-dimensional partial least squares discriminant analysis employing miRNA profiles for all 261 measured milk-derived miRNAs accounted for 21.6% of the variance in the data and achieved spatial separation of the four genotypes (wild-type, blue; T^288^S, purple; Exon 7, green; D^103^E, red).

**Figure 3:**
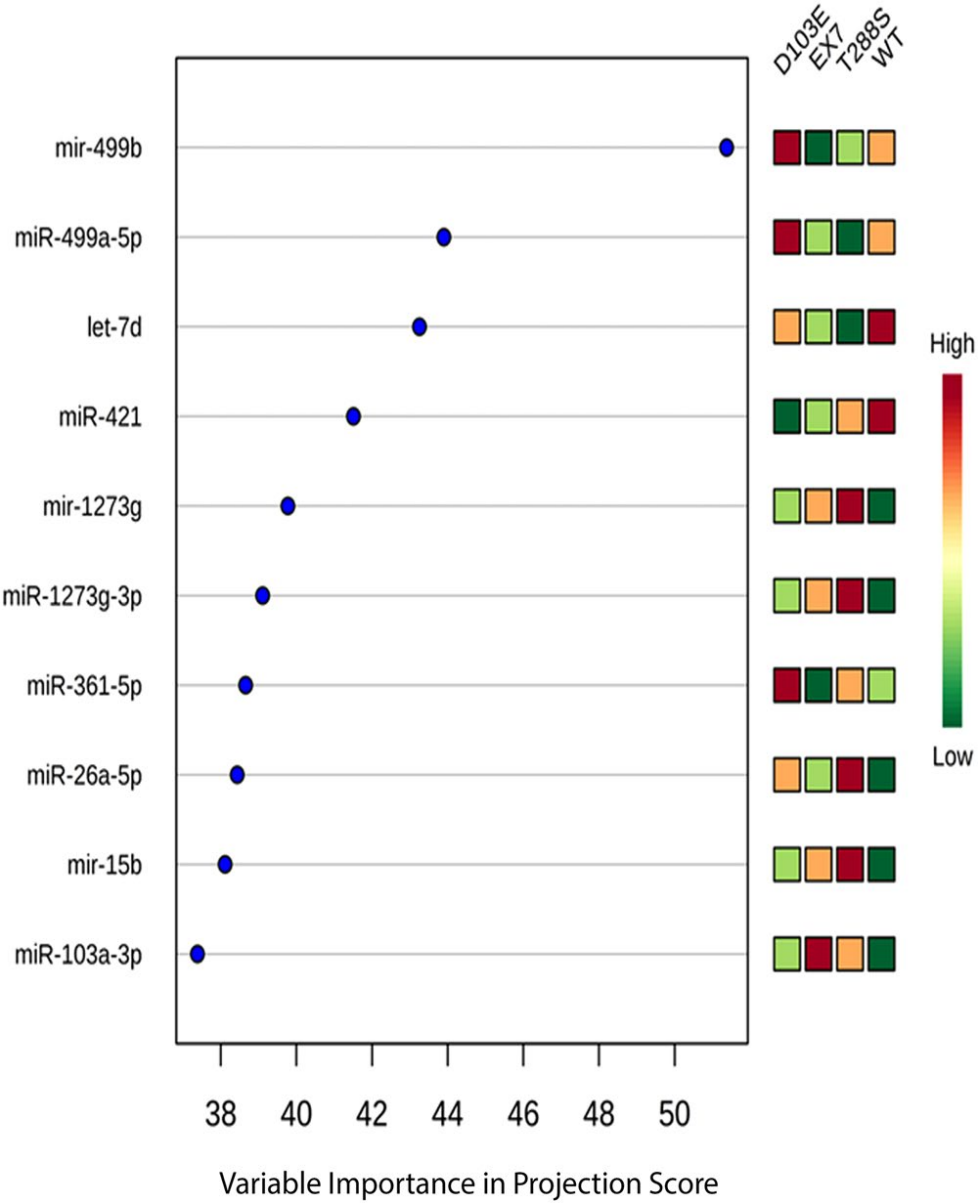
Ten milk-derived miRNAs most critical for group separation of ZnT2 genotypes. Variable importance of projection (VIP) score identifying the ten milk-derived miRNAs most important for separating the four *SLC30A2* genotypes. For each milk-derived miRNA, the relative abundance in each genotype was noted by shades of green (low abundance) or red (high abundance).

All samples (n = 4/genotype) passed RNA read-quality/quantity thresholds and were used for further analysis. Of the 2588 known human miRNAs interrogated for expression, 261 had robust concentrations (raw read counts > 10) in these milk samples (Supplementary File 1). Of those 261 miRNAs, 150 were mature miRNAs, and 111 were pre-miRNAs. A PLS-DA employing mature and pre-miRNA profiles across genotypes accounted for 21.6% of the variance in the data, and successfully separated the genotypes in two dimensional space (Fig. 2). The ten milk-derived miRNAs most crucial for this distinct separation were miR-103a-3p, miR-15b, miR-26a-5p, miR-361-5p, miR-1273g-3p, miR-1273g, miR-421, let-7d, miR-499a-5p, and miR-499b (Fig. 3). In samples from women with two normal ZnT2 alleles, miR-421 and let-7d had higher mean concentrations than all mutant ZnT2 genotypes. In contrast, miR-15b, miR-103a-3p, miR-26a-5p, miR-1273g, and miR-1273g-3p all had the lowest mean concentrations in samples from women with two normal ZnT2 alleles.

### Comparison of individual miRNAs across genotypes

**Figure 4:**
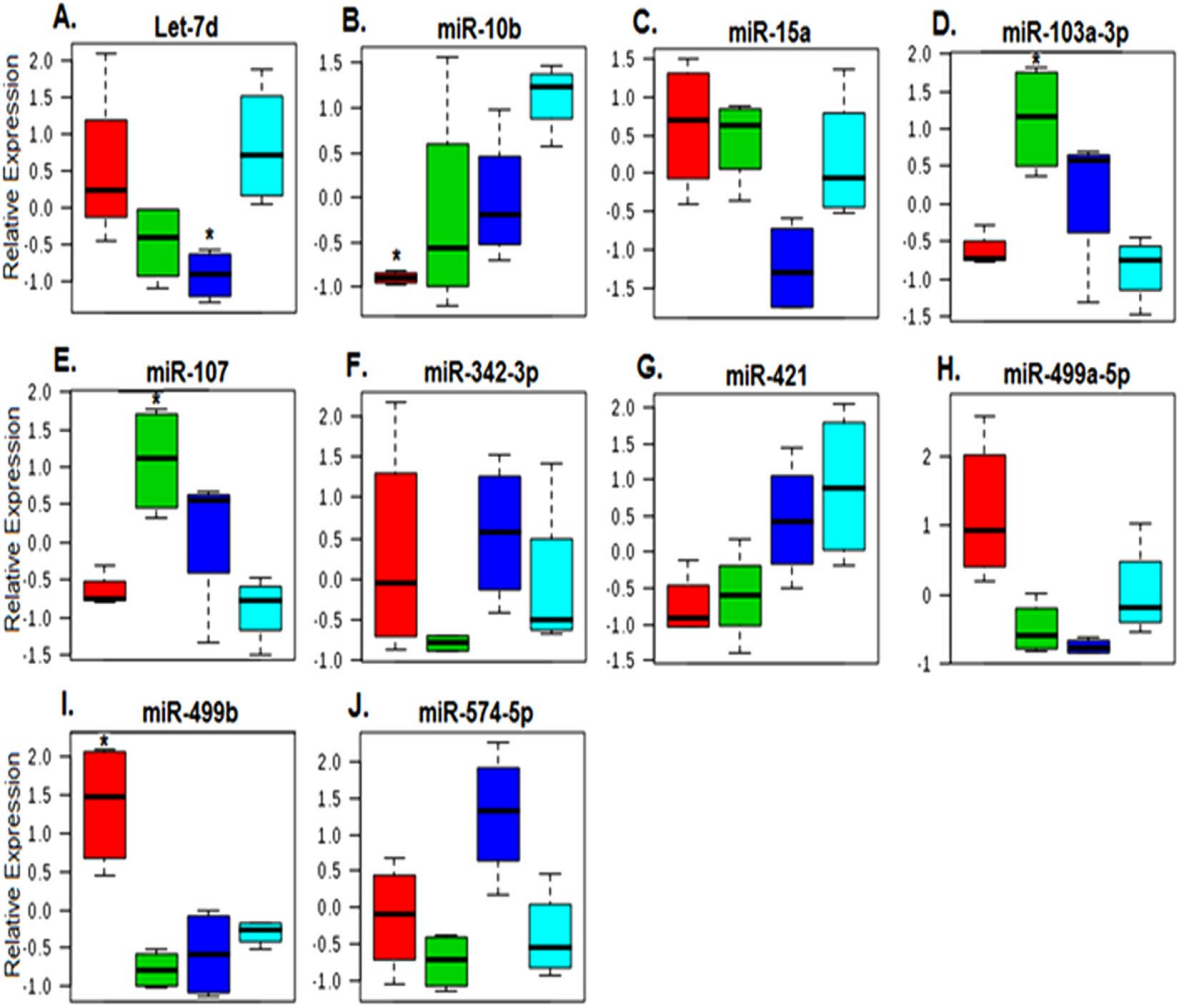
Ten milk-derived miRNAs displayed between-group differences using non-parametric analysis of variance. Box plots (A-J) represent mean relative expression ± SD for the ten milk-derived miRNAs most different among genotypes: D^103^E (red), Exon 7 (green), T^288^S (blue), and wild-type (light blue). Asterisks (*) denote samples with significant changes relative to wild-type controls on gene set analysis (p < 0.05).

Gene set analysis identified ten milk-derived miRNAs that showed nominal differences in expression (p < 0.05) between samples from women with two normal ZnT2 alleles and the other genotypes with Kruskal-Wallis (KW) testing (Fig. 4). Of the ten most critical miRNAs for spatial separation shown in Fig. 3, six of these were also identified with KW testing. Three miRNAs (miR-10b, miR-421, and let-7d) had lower expression in the variants relative to samples from women with two normal ZnT2 alleles, while two miRNAs (miR-103a-3p and miR-107) had generally higher expression in the variants relative to samples from women with two normal ZnT2 alleles. The remaining five miRNAs showed a mixed pattern of expression across genotypes. These miRNAs were used in a hierarchical clustering analysis, which again demonstrated complete separation of the genotypes. Women harboring the D^103^E variant clustered most-closely with women with two normal ZnT2 alleles, while women harboring the T^288^S and Exon 7 variants clustered most-closely with each other, mediated in-part by similar expression patterns in miR-107 and miR-103a-3p (Supplementary File 2).

### Functional analysis

**Figure 5:**
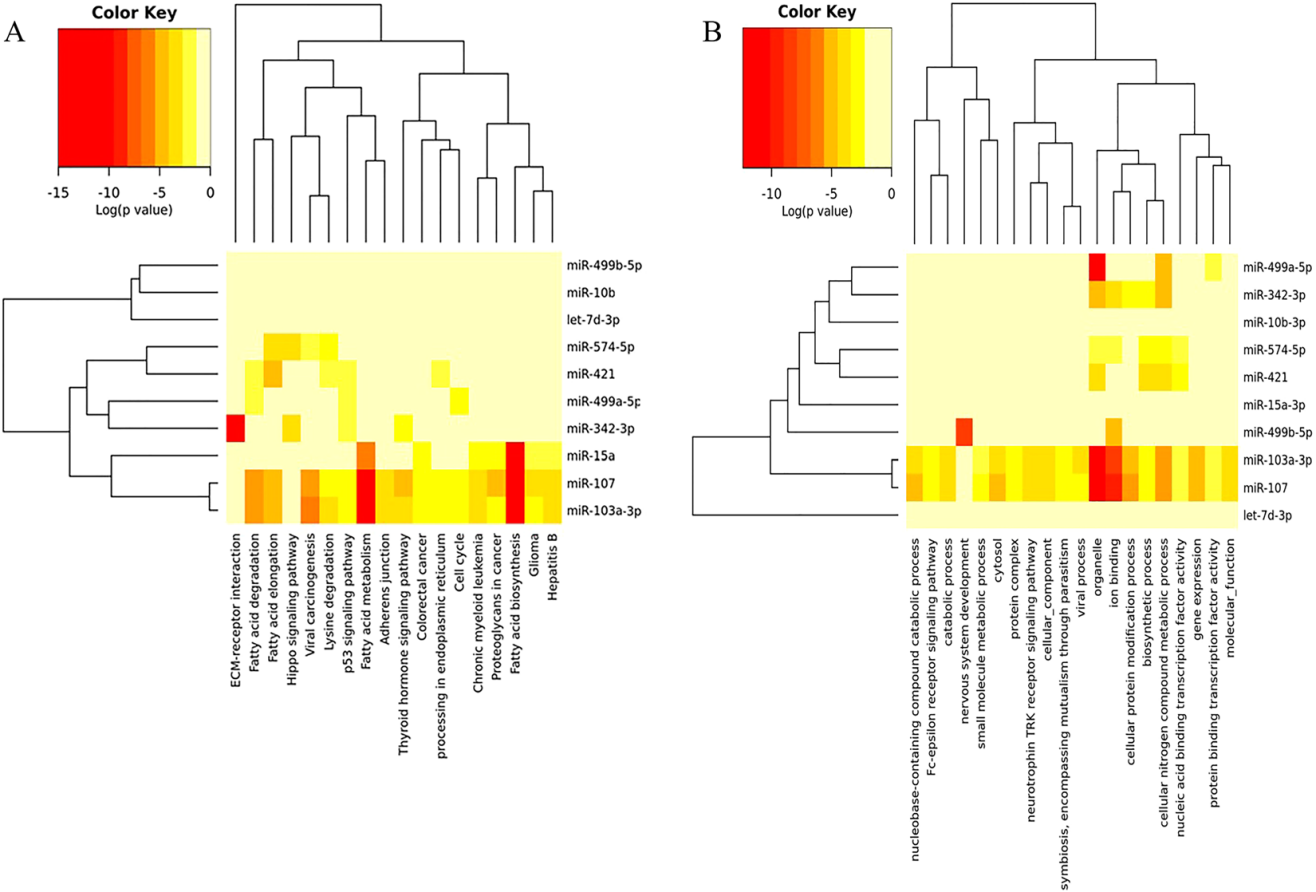
Hierarchical clustering of ten most crucial milk-derived miRNAs of interest based upon mRNA target function. (**a**) Gene targets for the ten miRNAs of interest were identified in DIANA using the Tarbase algorithm and KEGG Pathway functional representation was determined by number of gene targets per miRNA. Hierarchical clustering for the top-represented pathways was accomplished with Pearson Analysis. (**b**) Gene targets for the ten miRNAs of interest were identified in DIANA using the Tarbase algorithm and gene ontology (GO) functional representation was determined by number of gene targets per miRNA. Hierarchical clustering for the top-represented pathways was accomplished with Pearson Analysis.

**Figure 6:**
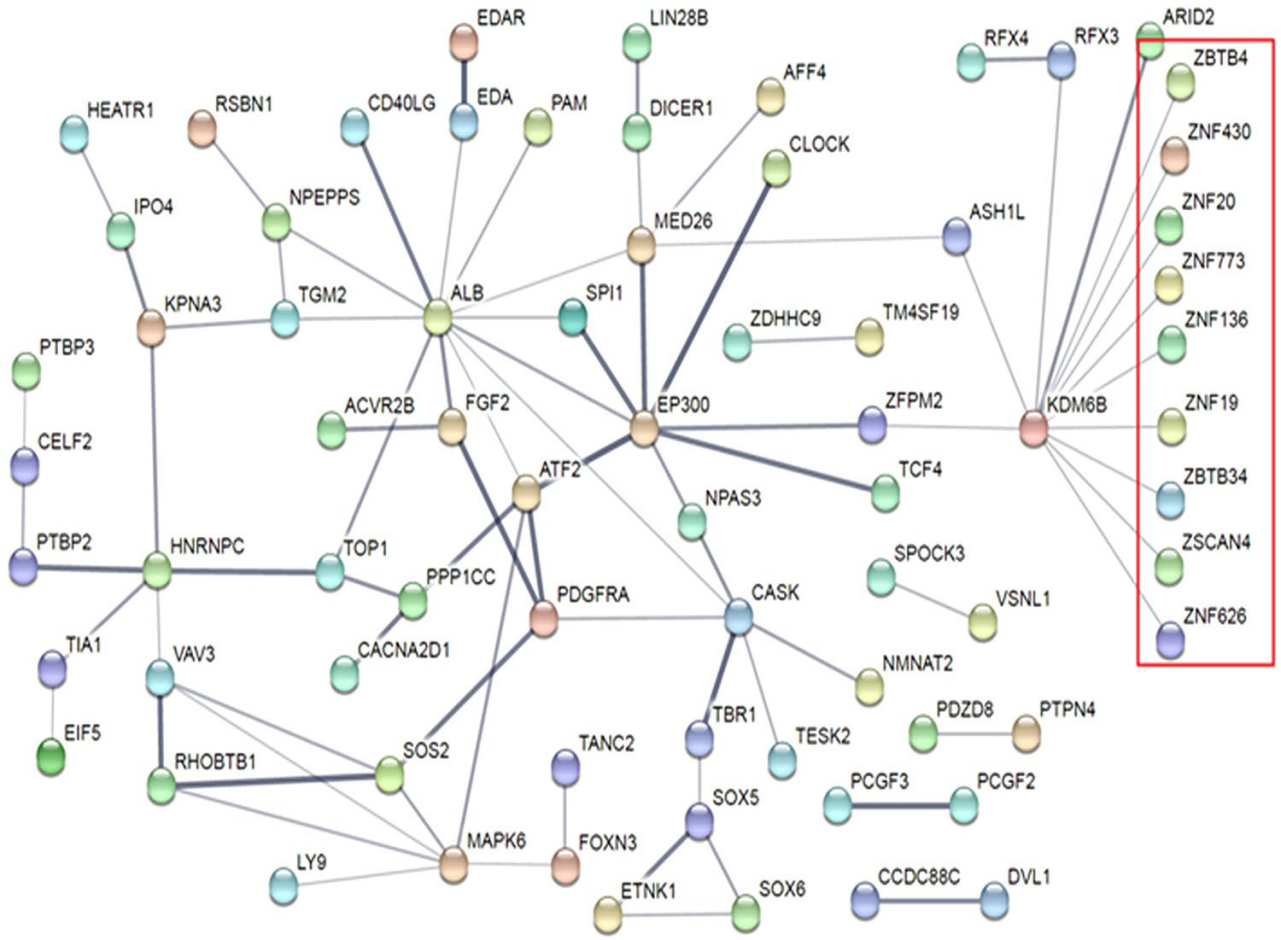
Protein interaction network for the high-fidelity mRNA targets of the ten most crucial milk-derived miRNAs of interest. High-fidelity (MicroT-CDS score > 0.99) mRNA targets of the ten miRNAs of interest were analyzed for protein-protein interactions using String v10 software. There was a greater number of protein-protein interactions within the network (p = 0.0267) than one would expect by chance alone. There were 28 gene ontology categories over-represented within the network (FDR < 0.05), many of which were related to cellular metabolism. The cluster highlighted in red contains a large amount of zinc-containing transcription factors.

The ten miRNAs with the largest differences across genotypes were chosen for functional analysis. These ten miRNAs had a total of 6875 predicted mRNA targets in DIANA mirPath v3. The mRNA targets were associated (FDR < 0.05) with 18 Kyoto Encyclopedia of Genes and Genomes (KEGG) pathways and 137 Gene Ontology (GO) categories (Supplementary File 3). The KEGG pathways affected by the largest number of miRNAs (3–5 miRNAs) included p53 signaling, fatty acid metabolism, ER processing, and cancers. The GO categories affected by the largest numbers of miRNAs (6–9 miRNAs) included organelle function, ion binding, RNA binding, biosynthetic processes, cellular component assembly, catabolic process, and response to stress. All predicted mRNA targets for each miRNA are shown in Supplementary File 4. The 20 mRNA targets with the highest target-prediction scores for each miRNA of interest (total = 139 mRNAs, because a portion of the 10 miRNAs of interest had <20 mRNA targets) were then categorized within each genotype based upon predicted direction of regulation. Several interesting targets included prolactin receptor (PRLR), which plays a vital role in mammary gland differentiation, lactation and milk production; vesicle associated membrane protein 7 (VAMP7), which plays a critical role in vesicle-mediated secretion; and sex-determining region Y-box 4 (SOX4), which is implicated in breast remodeling and tumorigenesis. Hierarchical clustering of mRNA target functions for these ten miRNAs of interest revealed putative functional relationships between miR-107 and miR-103a-3p, miR-421 and miR-574-5p, and miR-499a and miR-342 for KEGG pathways (Fig. 5a) and GO categories (Fig. 5b). These functional relationships were notable in light of the expression clustering changes observed in these miRNAs across genotypes (Fig. 4). Interrogation of the top 178 high-confidence mRNA targets with target scores ≥0.990 (Supplementary File 5) in String v10 demonstrated a significant protein interaction network (p = 0.0267) containing 136 nodes and 82 edges with a clustering coefficient of 0.798 (Fig. 6). Two key nodes identified were KDM6B (encoding lysine demethylase), which regulates cellular differentiation and development, tumorigenesis, inflammatory diseases, and EP300 (encoding histone acetyltransferase E1A binding protein 300), which regulates transcription via chromatin remodeling and is important in the processes of cell proliferation, differentiation, and co-activation of hypoxia-inducible factor 1 alpha. Analysis of the proteins in the network identified 28 biologic processes with significant (FDR < 0.05) enrichment (Supplementary File 6). Of note, 13 of the enriched GO processes were related to biosynthesis of nucleotides/gene expression and eight were related to regulation of macromolecule biosynthesis/metabolism.

### Breast milk analysis

**Figure 7:**
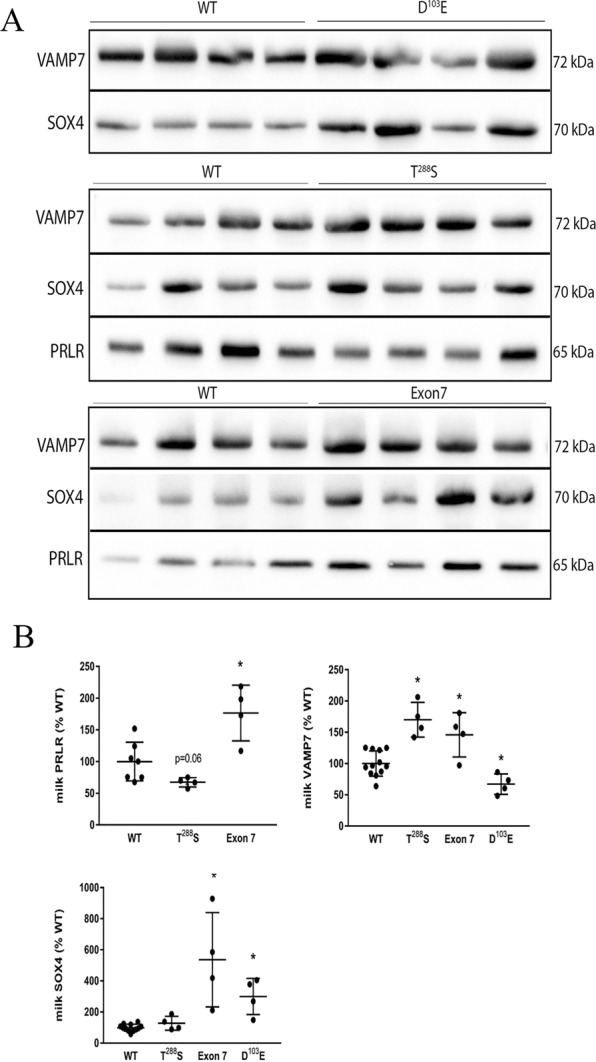
Relative abundance of predicted targets in breast milk. (**a**) Representative immunoblots of skimmed breast milk (5 uL) from women harboring two normal ZnT2 alleles (WT; n = 4) or common ZnT2 variants (T^288^S, D^103^E and Exon 7; n = 4/genotype). (**b**) Data represent mean ± SD each predicted target as a percent of the amount in women with two normal ZnT2 alleles. * Indicates a significant difference from women with two normal ZnT2 alleles, p < 0.05.

To provide direct evidence milk-derived miRNA profiles could successfully identify important mRNA targets, several proteins critical for mammary gland function predicted to be up- or down-regulated were selected for further analysis. We first measured the relative abundance of PRLR, VAMP7, and SOX4 in samples of skimmed breast milk (Fig. 7a,b). Consistent with our predictions, abundance of PRLR in breast milk tended to be lower in women who harbored T^288^S (p = 0.06), and was significantly higher in women who harbored Exon 7 (p < 0.01), relative to women who harbored two normal ZnT2 alleles. Abundance of VAMP7 in breast milk was significantly higher in women who harbored either T^288^S (p < 0.001) or Exon7 (p < 0.01), and was significantly lower in women who harbored D^103^E (p < 0.01) relative to women who harbored two normal ZnT2 alleles, consistent with our predictions based on milk-derived miRNA levels. However, our prediction of SOX4 abundance in breast milk was inconsistent. While we did find that SOX4 was significantly higher in women who harbored Exon7 (p < 0.001), we found no difference in women who harbored T^288^S (p = 0.1), and SOX4 was significantly higher in women who harbored D^103^E (p < 0.0001) relative to women who harbored two normal ZnT2 alleles.

### Transfection of cultured MECs

**Figure 8:**
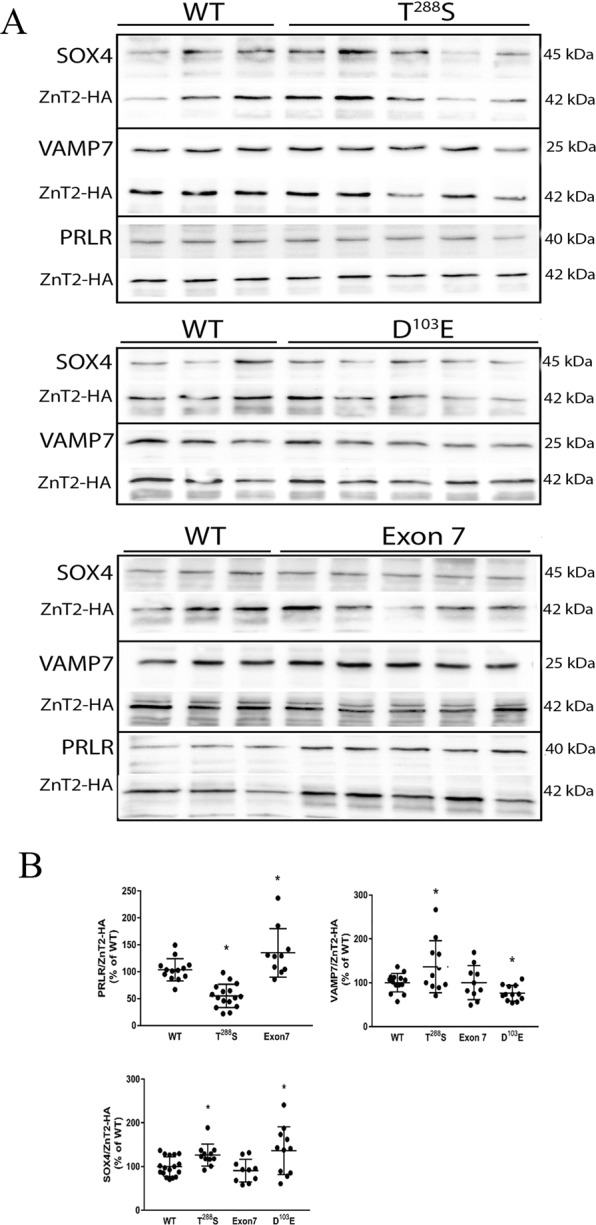
Relative abundance of predicted targets in mammary epithelial cells expressing each ZnT2 variant. (**a**) Representative immunoblots of total cell protein extracts (20 ug) from MECs expressing wild-type ZnT2 (WT; n = 3) or common ZnT2 variants (T^288^S, D^103^E and Exon 7, n = 5/mutant). (**b**) Data represent mean ± SD of each predicted target normalized to ZnT2-HA, and expressed relative to MECs expressing WT ZnT2. *Indicates a significant difference from WT, p < 0.05.

To directly evaluate the effect of each ZnT2 variant on abundance of our predicted targets, we expressed each variant in MECs and measured the expression of PRLR, VAMP7, and SOX4 (Fig. 8a,b) relative to MECs expressing wild-type (WT) ZnT2. Milk-derived miRNA profiles predicted that MECs expressing T^288^S would have lower abundance of PRLR and greater abundance of VAMP7 and SOX4, which was confirmed by immunoblotting (p < 0.0001, p < 0.05, p < 0.01, respectively). Milk-derived miRNA profiles predicted MECs expressing Exon7 would have greater abundance of PRLR, VAMP7 and SOX4. While changes in PRLR abundance were confirmed by immunoblotting (p < 0.05), there was no significant effect of expressing Exon 7 on VAMP7 or SOX4 abundance. Milk-derived miRNA profiles predicted MECs expressing D^103^E would have lower abundance in VAMP7 and SOX4. While VAMP7 levels were consistent with our prediction (p < 0.05), SOX4 levels were significantly greater compared to MECs expressing WT ZnT2 (p < 0.05), which was consistent with our observations in breast milk samples.

## Discussion

Human milk is one of the richest sources of miRNAs^30^, and an intriguing hypothesis is that milk-derived miRNAs secreted from MECs reflect mammary gland function during lactation^6^. Previous studies in cultured MECs and preclinical mouse models document loss or impaired ZnT2 function compromises MEC function. Here we hypothesized women who harbor common non-synonymous variants in ZnT2 have unique differences in their milk-derived miRNA profile, which allow us to predict important functions that may be impaired. One key finding was the identification of two nodes, at KDM6B and EP300, which regulate transcription via chromatin remodeling and are important in the processes of cell proliferation and differentiation. These genes play important roles in defining the methylome, therefore harboring ZnT2 variants could affect the global pattern of mammary gland gene expression during development (puberty), expansion and differentiation (pregnancy and lactation), and extensive and coordinated remodeling (involution). This implicates genetic variation in *SLC30A2* as a driver of mammary gland dysfunction. Furthermore, miRNA profiling allowed us to identify critical cell functions and gene networks that may be disrupted in women who harbor specific ZnT2 variants.

Women harboring T^288^S, the most common variant we previously identified (16% frequency)^23^, had biomarkers of oxidative and ER stress evident in their breast milk^29^ and an elevated milk Na/K ratio^23^, strongly suggesting breast dysfunction. *In vitro* studies determined T^288^S is mislocalized to the ER and lysosomes, and leads to accumulation of ER and lysosomal zinc, and activation of STAT3 signaling; however, the molecular and clinical implications of these observations are not currently understood. To identify molecular pathways involved in cellular dysfunction, we profiled milk-derived miRNAs and found that the overall profile from women harboring T^288^S suggests a robust cellular response to defects in ZnT2 function. Four miRNAs tended to be lower (let-7d, miR-499a, miR-15a, and miR-10b) while three miRNAs tended to be higher (miR-574-5p, miR-103a-3p and miR-107) relative to women with two wild-type alleles. Four of these have previously been shown to affect breast cell function. miR-15a is a member of the proapoptotic miRNA cluster (miR-15a/16) that activates caspase 3/7 and apoptosis^31^. Thus, downregulation in the context of lactation might serve to interfere with cellular turnover and impair alveolar development. Moreover, all three miRNAs upregulated in women who harbor T^288^S have been implicated in cell motility and epithelial-to-mesenchymal transition in breast cancer^32,33^. While it is intriguing to speculate that milk-derived miRNAs may be useful as markers for breast cancer risk, further research is required to address this possibility. Two of the numerous mRNA targets predicted to be upregulated include VAMP7 and SOX4. Interestingly, we found we could easily detect VAMP7 by immunoblotting using 5 µL of breast milk. Moreover, abundance of VAMP7 relative to women who harbored two normal alleles was significantly higher as predicted, and this was a direct effect of T^288^S expression in MECs. VAMP7 is a vesicular SNARE protein critical for autophagy and nutrient sensing, thus zinc accumulation into lysosomes may lead to alterations in lysosome biogenesis, nutrient sensing and intracellular energy metabolism and may contribute to breast dysfunction in women who harbor T^288^S. Armed with this novel information, targeted studies can be conducted to explore this possibility. While we were also able to detect SOX4 in breast milk, there was no detectible difference in the relative amount of SOX4 in the breast milk of women harboring different genotypes. However, consistent with our prediction, T^288^S expression in MECs increased SOX4 abundance. SOX4 is a transcription factor that mediates transcriptional response to Wnt signaling regulating numerous gene networks involved in miRNA processing, transcriptional regulation, the TGFβ, Wnt, Hedgehog, and Notch pathways, and growth factor signaling^34^. In MECs, SOX4 induces TGFβ and drives mesenchymal transition^35^, which may be particularly deleterious to maintaining a secretory phenotype. Thus our identification of SOX4 as a novel molecular target provides numerous novel avenues for further exploration. A gene indispensable for lactation and predicted to be downregulated in women harboring T^288^S is PRLR, which was confirmed in both breast milk and in MECs expressing T^288^S. Prolactin signaling through its cognate receptor (reviewed in^36^) is essential for normal mammary gland function during lactation, thus down-regulation of this protein would have critical implications for mammary gland differentiation, milk production and secretion during lactation. Collectively, this suggests that of the three ZnT2 variants explored here, women who harbor T^288^S may be at greatest risk for poor lactation performance due to alterations in cellular turnover, alveolar development, nutrient processing, and numerous pathways associated with VAMP7, SOX4 and PRLR signaling. As mentioned previously, women harboring T^288^S have significantly higher milk sodium concentrations compared to women with two wild-type alleles^23^. High milk sodium levels result from the disorganization of tight junctions that leads to the loss of barrier function and paracellular influx of sodium. Such disorganization has been described in women who deliver preterm^25,37^, women with mastitis^27^, and as a consequence of mammary gland remodeling during involution^26^. We recently showed expression of T^288^S in cultured MECs leads to defects in barrier function^29^. This is consistent with results herein which suggest that expression of critical mRNAs involved in oxidative stress response (*DNAJA1*, *APEX1*), polarity (*ARHGAP1*), differentiation (*PRLR*, *RREB1*), and cytoskeletal arrangement (*PLXNA2*, *PFN1*, *KIAA0754*) may be downregulated in women who harbor T^288^S. Further targeted studies will be necessary to investigate these potential pathways.

The second most common ZnT2 variant we previously identified was D^103^E (9% frequency)^23^. While effects on milk production were not addressed, the milk Na/K ratio was also elevated, suggesting breast dysfunction. Moreover, studies in MECs *in vitro* found D^103^E was also mislocalized to the ER, late endosome and lysosomes; however, unlike T^288^S, profound loss-of-function and consequences on cell cycle were documented. It is possible the inability to transport zinc causes extreme cellular dysfunction; however, the molecular pathways affected remain to be identified. We found the profile of milk-derived miRNAs was also distinct from women with two wild-type alleles; two miRNAs tended to be higher (miR-499a and miR-499b) and predicted the downregulation of numerous mRNAs including VAMP7. This prediction was confirmed in breast milk and MECs expressing the mutant, suggesting decreased lysosome biogenesis, nutrient sensing and intracellular energy metabolism. Moreover, two miRNAs tended to be lower (miR-421 and miR-10b) which predicted upregulation of numerous mRNAs including SOX4, which was also confirmed in breast milk and MECs expressing the mutant. We previously showed that MECs expressing D^103^E had profound changes in cell cycle^23^. Here we propose that increased expression of SOX4, which regulates cell cycle^38^, may be at least partially responsible for these observations. Moreover, upregulation of SOX4 has been associated with breast cancer and proposed to be the master regulator of epithelial-to-mesenchymal transition^34^, which again suggests mutants in ZnT2 may impair the secretory phenotype, and may affect risk for breast disease.

The least understood ZnT2 variant we previously identified is Exon 7 (7% frequency)^23^. The composition of this “variant” is quite complex, as it consists of numerous compound substitutions in the C-terminus. Milk-derived miRNA profiling provided insight as to potential pathways to explore. In women expressing Exon 7, five miRNAs tended to be lower (let-7d, miR-499a, miR-499b, miR-342 and miR-10b) and two miRNAs tended to be higher (miR-103a-3p and miR-107) compared to women with two wild-type alleles. As a result, abundance of PRLR, VAMP7 and SOX4 were all predicted to be upregulated, and this was confirmed in the breast milk of women harboring Exon 7. However, while we were able to confirm that PRLR expression was indeed upregulated in MECs expressing Exon 7, we found no difference in VAMP7 or SOX4. This discrepancy is not currently understood; however, unknown genotypic and/or phenotypic differences may contribute. For example, the mutant may lead to the upregulation of transcriptional suppressors or factors that reduce VAMP7 or SOX4 stability that remain to be defined. This highlights the need to carefully characterize this unusual ZnT2 variant and empirically assess the cellular consequences of milk-derived miRNAs *in vitro*. A major difference between women harboring Exon 7 and T^288^S was the expression of PRLR, which was upregulated in women who harbor Exon 7. A consequence of upregulated PRLR might be enhanced milk production or secretion. In fact, it is interesting to note ZnT2 and β-casein are regulated by PRL signaling, and women who harbor Exon 7 have higher zinc levels in their milk^23^ and elevated β-casein levels (data not shown). However, it is also possible that despite enhanced PRLR abundance, defects in PRLR localization or signaling may exist that impair lactation. Clearly, further studies are needed to understand effects of this variant on zinc transport, and to understand the molecular pathways affected by Exon 7.

Human milk provides a complex array of nutrients and bioactive factors that protect the infant against infection and confers both short and long-term health benefits; however, the mechanisms behind why those benefits are achieved are not well-understood. Many of these factors, including milk-derived miRNAs may act as key developmental regulators of the gastrointestinal tract, which is dynamically regulated by epigenetic modifications and transcription factors, influencing gene expression in intestinal cells throughout the crypt-villus axis. Milk-derived miRNAs withstand the harsh conditions of the gut and are taken up by human intestinal and vascular endothelial cells via endocytosis^39^. In this manner, nutritional miRNA can modify gene expression and promote proliferation in intestinal cells *in vitro* and *in vivo*^40,41^. The framework for understanding potential roles for milk-derived miRNAs on postnatal growth and development has been reviewed elsewhere^7^; however, extensive research is required to better understand the role of milk-derived miRNAs in regulating gastrointestinal and immune function in the neonate. Intriguingly, several miRNAs that were enriched in milk samples from women harboring genetic variation in *SLC30A2* (miR-103, miR-107, miR-15a and miR-574) regulate developmental processes. For example, miR-103, miR-107, and miR-15a/b can regulate glucose tolerance and insulin-sensitivity^42^, suggesting a potential role for modulating glucose homeostasis and perhaps growth or body composition in infants. Recent studies have shown that miR-103 and miR-107 regulate cyclin-dependent kinase 5 regulatory subunit 1, the main activation subunit of cyclin-dependent kinase 5 that plays a fundamental role in brain development and function^43^. The miRNA pair also target death-associated protein kinase and Krüppel-like factor 4, which affects cell motility, cell-matrix adhesion, cell-cell adhesion and epithelial marker expression, pathways vital to gastrointestinal development^44^. Moreover, miR-574 represses SMAD4^45^ and protein tyrosine phosphatase receptor type U expression^46^, both of which are critical for regulating gastrointestinal development through BMP/SMAD and β-catenin signaling. Intriguingly, numerous mRNAs involved in the GO functions of viral life cycle, viral process, viral transcription, positive regulation of viral genome, and virion assembly were targeted by the ten miRNAs altered among ZnT2 genotypes. Thus, disruptions in the ZnT2 locus may have additional, critical influences on the developing immune response in the neonate.

How variants in ZnT2 elicit these effects is not understood. This may result from aberrant transport of zinc (or lack thereof) into specific sub-cellular pools^47,48^ that affects key biological processes such as transcription or miRNA biogenesis. Alternatively, recent studies suggest that several ZnT proteins may form stable heterodimers that are localized to distinct intracellular compartments, some of which are completely different from their homodimer localization^49,50^. While to our knowledge ZnT heterodimerization has only been shown in transfected cell systems, these studies suggest an intriguing level of zinc transporter complementation that needs further exploration. Moreover, the idea that variants in ZnT2 may alter heterodimer location and function is an additional avenue that warrants investigation, given how common non-synonymous variants in ZnT2 are in the population^22^.

There are several limitations of this study that should be noted. First, this study is limited by its small sample size. Although, the distinct separation in miRNA profiles observed between genotypes reveals robust differences, it is possible that a larger sample size would have detected further significant differences between genotypes and identified additional discriminatory miRNAs. Thus, we chose to focus only on the top ten miRNAs that were significantly different between at least one ZnT2 variant and two normal allele controls, and then also carefully characterized levels of predicted targets both in breast milk and in cultured MECs expressing the mutants. Another limitation is the lack of clinical data to correlate milk-derived miRNA profiles with measures of lactation performance in these subjects. This cross-sectional study was conducted at ~4 months of lactation, and milk energy density (the concentrations of protein, fat, lactose and citrate) was not different from women with two normal alleles. However, our recent report describing markers in the breast milk of women expressing T^288^S combined with *in vitro* studies in MECs transfected to express this ZnT2 variant^29^ is consistent with impaired lactation performance. Nevertheless, further studies that measure milk volume and ejection, and assess cellular function in MECs freshly isolated from breast milk are required to fully appreciate the consequence of expressing ZnT2 variants on lactation performance and breast health in women. In addition, the extent of breastfeeding (i.e., exclusive, predominant, partial, etc.) was not documented in the women in our study, thus it is possible that at least some of the variability in milk-derived miRNA expression may have been due to these differences. Finally, our isolation of miRNAs from the milk lipid fraction did not permit a focus on miRNAs contained specifically within exosomes. Our approach to miRNA extraction from the lipid fraction was informed by our prior study of human breastmilk miRNA, demonstrating over 10-fold enrichment in lipid (465 individual miRNA features) relative to skim milk (32 miRNA features)^11^, and this approach was originally informed by studies of human breastmilk miRNA by Alsaweed and colleagues^6,30^. This seems in contrast to studies of miRNAs in bovine milk where the milk lipid fraction contains predominantly milk fat globules and the bulk of miRNAs are found in exosomes in the skimmed fraction^51^. Characterization of exosome-specific miRNAs might identify miRNAs most relevant to intracellular signaling, or most likely to survive maternal-infant transfer, as exosomes are resistant to digestion^52,53^ and thus miRNAs contained within may have biological activity in the newborn^54^. However, specifically profiling exosomal miRNAs could exclude important protein-bound or cellular miRNAs that are relevant to ZnT2 variant status, which was of primary interest to the current study. However further studies are needed, because as a vesicular zinc transporter, specific analysis of the exosomal fraction may identify novel roles for ZnT2 in exosome zinc accumulation^55^, biogenesis, cargo loading, or secretion^56^.

In summary, our results provide evidence milk-derived miRNA profiles can be used to identify novel molecular pathways that underscore breast function during lactation in women. Moreover, our study provides evidence that women who harbor non-synonymous variants in *SLC30A2* may be at risk for poor lactation performance, which given how common variants in *SLC30A2* are^22^, this should be further explored. Intriguingly, our results also suggest that there may be developmental consequences in infants who nurse from mothers expressing ZnT2 variants that warrant further investigation.

## Methods

### Milk collection

The study was approved by the Institutional Review Board of the Pennsylvania State University and informed consent was obtained form study participants. All methods were performed in accordance with the relevant guidelines and regulations. Results of genotyping and milk zinc and sodium levels were previously reported^23^. Briefly, inclusion criteria included healthy women between 18 and 40 years old who were breastfeeding one healthy infant at ~4 months post-partum. Exclusion criteria included: pre-term birth (<37 week gestation), multiples, smokers, or non-English speaking. Written consent was obtained from all subjects. A sample of breast milk (~20 mL) was taken from a complete collection (fore and hind milk) obtained from one breast in the morning and frozen at −20 °C until analysis.

### Identification of genetic variants

Sequencing of *SLC30A2* was performed and genetic variants were identified, confirmed, and reported previously^23^. Milk-derived miRNAs from women harboring the three most common genetic variants (D^103^E, T^288^S, Exon 7) were compared with women who harbored two wild-type *SLC30A2* alleles (n = 4/genotype).

### RNA extraction

Milk samples were flash-thawed and centrifuged for 20 min at 4 °C at 800 rpm to isolate the lipid fraction. The lipid fraction was chosen due to its high miRNA content relative to the skimmed milk. RNA extraction^6,11,30^ was accomplished using the Norgen Circulating and Exosomal RNA Purification Kit (Norgen Biotek, Ontario, Canada) according to the manufacturer’s protocol. This kit allows for isolation of both free and lipid-bound RNA species. For each sample, the lipid fraction (50 μL) was diluted to 1 mL with phosphate-buffered saline in RNAse-free tubes for extraction. Exosomes were eluted with 50 μL of elution buffer and the column was centrifuged at 2,000 rpm for 2 min followed by a second centrifugation at 14,000 rpm for 3 min. A second elution was repeated with the initial flow-through. The final RNA concentration was determined using a Nanodrop spectrophotometer (Nanodrop ND-1000, Thermo Scientific) and the extracted RNA was stored at −80 °C prior to sequencing.

### RNA sequencing and alignment

The yield and quality of the RNA was assessed with an Agilent 2100 Bioanalyzer prior to library construction with the NEXTflex Small RNA-Seq Kit v3 (Bio Scientific; Austin, Texas). Multiplexed samples were run on an Illumina HiSeq. 2500 Instrument at a targeted depth of one million reads per sample. FastQ outputs were clipped, trimmed and filtered to a maximum read length of 30 base pairs using the FASTX Toolkit Module in Mobaxterm. Reads were aligned to the hg38 build of the human genome in Partek Flow (Partek; St. Louis, Missouri) using the SHRIPM2 aligner. Total counts for both pre- and mature-miRNAs were quantified using miRBase microRNAs v21 and read counts were normalized across samples using a trimmed mean of M-values (TMM) method. Samples with less than 30,000 human miRNA alignments were excluded from the analysis. Only those miRNAs with raw read counts >10 in at least 25% of samples were evaluated in the differential expression analysis.

### Statistical analysis

A multi-model approach employing gene set analysis (GSA) in Partek Flow was first used to identify individual miRNAs with differential expression patterns between wild-type and T^288^S, Exon 7, or D^103^E samples, respectively. Expression patterns of mature and pre-miRNAs across genotypes were visualized in Metaboanalyst (http://www.metaboanalyst.ca/) with non-parametric Kruskall-Wallis (KW) testing. All miRNAs were subjected to Benjamini Hochberg false detection rate (FDR) correction for multiple comparisons. Fold-change in miRNA levels between groups was reported as a log2 value. Spatial relationships between the total miRNA profiles for each group were examined with a two-dimensional partial least squares discriminant analysis (PLS-DA). The miRNAs most crucial for separation of genotypes were identified by mean Variable Importance in Projection (VIP) score, a weighted sum of squares of the PLS loading curve which takes into account explained dimensional variation. Hierarchical clustering was performed for the four genotypes using a Pearson distance measure of the top ten miRNAs on KW testing with a complete clustering algorithm.

### Functional annotation

The miRNAs with differential expression across genotypes underwent functional annotation analysis in DIANA mirPath v3 online software (http://snf-515788.vm.okeanos.grnet.gr/). The Tarbase v7.0 algorithm was used to identify mRNA targets for each miRNA of interest. Tarbase employs a manually curated database of >500,000 miRNA:gene interactions from published experimental data utilizing 356 different cell types from 24 species. DIANA mirPath was used to identify Kyoto Encyclopedia of Genes and Genomes (KEGG) pathways and gene ontology (GO) categories with significant enrichment (FDR < 0.05) for the miRNAs that were different among genotypes. Functional enrichment was determined with Fisher’s Exact Tests, which used a 2 × 2 table to compare the ratio of identified mRNA targets to the number of targets expected by chance alone. Relationships between individual miRNAs and KEGG and GO variables were visualized with hierarchical clustering. A list of 178 high confidence mRNA targets for the ten miRNAs of interest (DIANA microT-CDS score ≥ 0.990) was interrogated for protein-protein interaction networks using moderate stringency settings (interaction score > 0.40) in String v10 software (http://string-db.org). String uses annotated online databases to identify and evaluate biochemical interactions between lists of mRNA-protein products. Biological pathways significantly over-represented in the high confidence target gene sets were reported (FDR < 0.05). Finally, a list of 24 zinc transporter genes from the *SLC30A* and *SLC39A* families was interrogated for predicted interaction with the ten miRNAs of interest using miRDB online software (http://www.mirdb.org).

### Generation of plasmid DNA construct

The full-length wild-type and variant forms of ZnT2 protein tagged with a C-terminal tandem hemagglutinin were generated as previously described^23^.

### Cell culture and *In Vitro* Expression of ZnT2 variants

Mouse MECs (HC11 cells) were a gift from Dr. Jeffery Rosen (Baylor College of Medicine, Houston, TX), used with permission of Dr. Bernd Groner (Institute for Biomedical Research, Frankford, Germany) and maintained in growth medium as previously described^23^. Cells were plated in antibiotic-free growth medium in 6-well plates and transiently transfected with 4 μg of either wild-type or variant plasmid using Lipofectamine 2000 (Invitrogen) for 5 h according to manufacturer’s instruction. Transfected MECs were used for experiments 10–24 h later. Transfections were verified by immunoblotting with anti-HA antibody as described below.

### Immunoblotting

Skimmed milk (5 µL) or MEC lysates (20 µg of protein) were prepared in Laemmli sample buffer containing 100 mM dithiothreitol (DTT), electrophoresed and immunoblotted as previously described^29^. The following antibodies were used: anti-PRLR (1:1000, Abcam), anti-VAMP7 (1:1000, ThermoFisher Scientific), anti-SOX4 (1:1000, ThermoFisher Scientific), and anti-HA (1:1000; Roche Applied Scientific). Antibodies were detected with horseradish peroxidase-conjugated anti- rabbit or anti-mouse IgG (GE Healthcare). Membranes were stripped before re-probing with anti-HA as a normalization control. Protein was detected with SuperSignal Femto Chemiluminescent Detection System (Pierce) and imaged using digital imaging (BioRad). Band signal intensity was quantified using ImageStudio software (LICOR).

## Supplementary information

Supp data 1

Supp data 2

Supp data 3

Supp data 4

Supp data 5

Supp data 6

Supplementary Info
