# Supplementary material for: Milk-derived miRNA profiles elucidate molecular pathways that underlie breast dysfunction in women with common genetic variants in SLC30A2
Source: Sci Rep. 2019 Sep 3;9:12686. doi: 10.1038/s41598-019-48987-4 (PMC6722070; doi:10.1038/s41598-019-48987-4)
Supplement: Supplementary file 2 — Supp data 2 [file 41598_2019_48987_MOESM2_ESM.docx]

Milk-derived miRNA profiles elucidate molecular pathways that underlie breast dysfunction in women with common genetic variants in *SLC30A2*

*Shannon L. Kelleher^1,2,3^, Annie Gagnon^1^, Olivia C. Rivera^2,3^, Steven D. Hicks^4^, Molly C. Carney^4^, and Samina Alam^3^

^1^Department of Biomedical and Nutritional Sciences, University of Massachusetts Lowell, Lowell, Massachusetts, United States of America

^2^Department of Cellular and Molecular Physiology, Penn State Hershey College of Medicine, Hershey, Pennsylvania, United States of America

^3^Department of Surgery, Penn State Hershey College of Medicine, Hershey, Pennsylvania, United States of America

^4^Department of Pediatrics, Penn State Hershey College of Medicine, Hershey, Pennsylvania, United States of America

*Corresponding author

Shannon L Kelleher, PhD

The University of Massachusetts Lowell

883 Broadway Street, Dugan Hall 110R

Lowell, MA 01852

Phone: 978-934-3527

E-mail: shannon_kelleher@uml.edu

ORCID ID: orcid.org/0000-0003-0613-6294


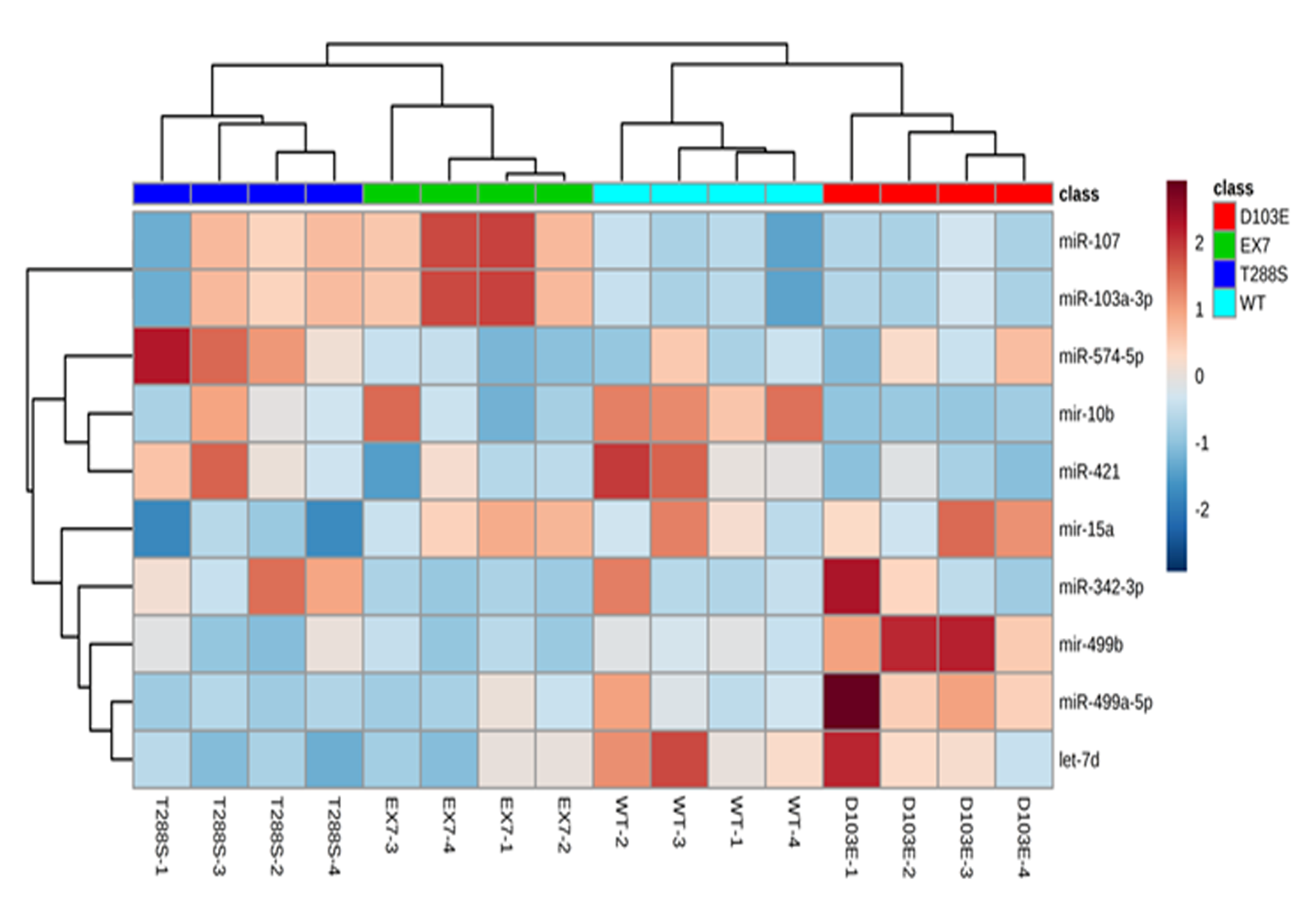
Supplementary File 2

Hierarchical clustering analysis of samples employing ten most crucial miRNAs of interest.

Hierarchical clustering of the ten milk-derived miRNAs with the most significant changes across the four genotypes showed distinct clustering of Exon 7 and T^288^S from wild-type and D^103^E. Colored scale values indicate average Z score of normalized abundance for each miRNA.
