# Supplementary material for: Milk-derived miRNA profiles elucidate molecular pathways that underlie breast dysfunction in women with common genetic variants in SLC30A2
Source: Sci Rep. 2019 Sep 3;9:12686. doi: 10.1038/s41598-019-48987-4 (PMC6722070; doi:10.1038/s41598-019-48987-4)
Supplement: Supplementary file 7 — Supplementary Info [file 41598_2019_48987_MOESM7_ESM.pdf]

Milk-derived miRNA profiles elucidate molecular pathways that underlie breast dysfunction in women with common genetic variants in *SLC30A2*

*Shannon L. Kelleher[1,2,3], Annie Gagnon[1], Olivia C. Rivera[2,3], Steven D. Hicks[4], Molly C. Carney[4], and Samina Alam[3]

[1]Department of Biomedical and Nutritional Sciences, University of Massachusetts Lowell, Lowell, Massachusetts, United States of America
[2]Department of Cellular and Molecular Physiology, Penn State Hershey College of Medicine, Hershey, Pennsylvania, United States of America
[3]Department of Surgery, Penn State Hershey College of Medicine, Hershey, Pennsylvania, United States of America
[4]Department of Pediatrics, Penn State Hershey College of Medicine, Hershey, Pennsylvania, United States of America

VAMP7 Milk

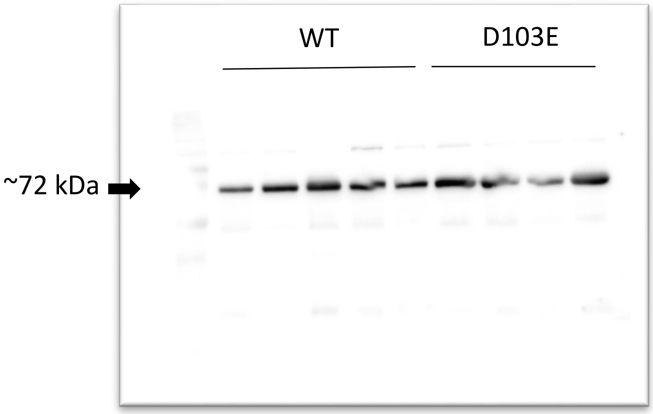

VAMP7 Milk

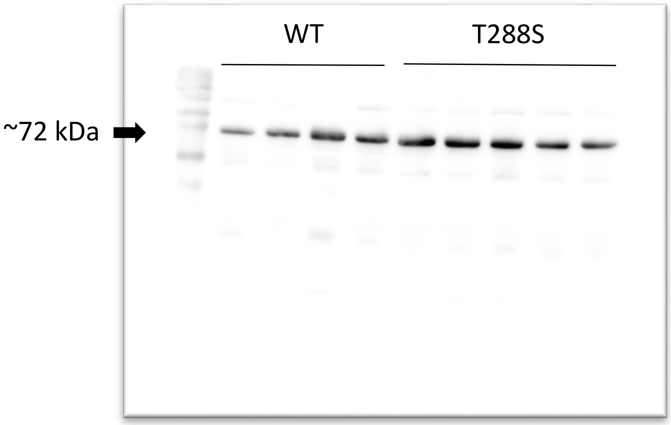

VAMP7 Milk

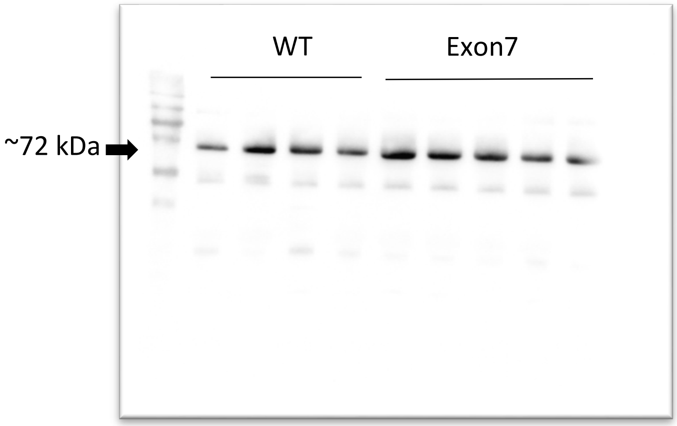

SOX4 Milk

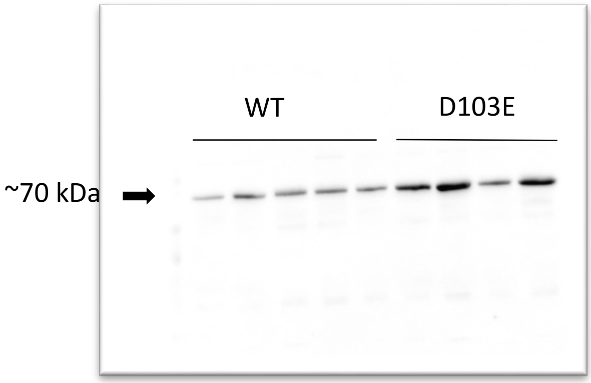

SOX4 Milk

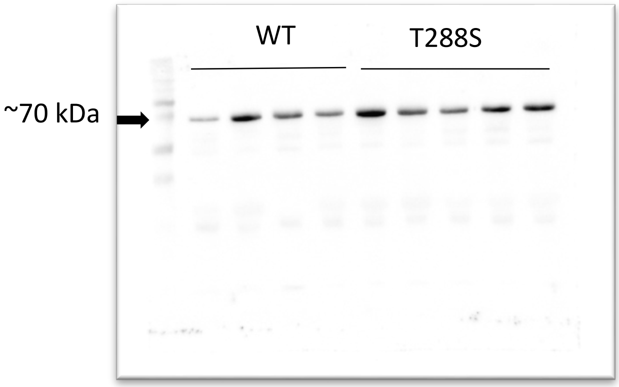

SOX4 Milk

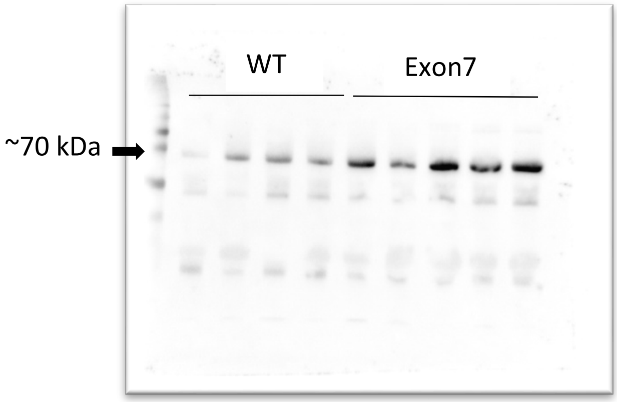

PRLR Milk

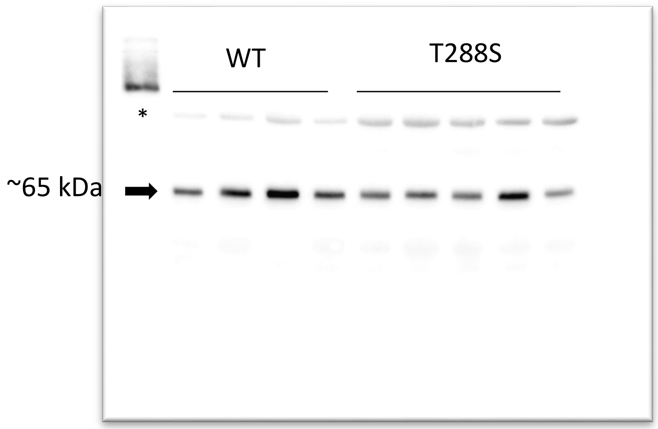

PRLR Milk

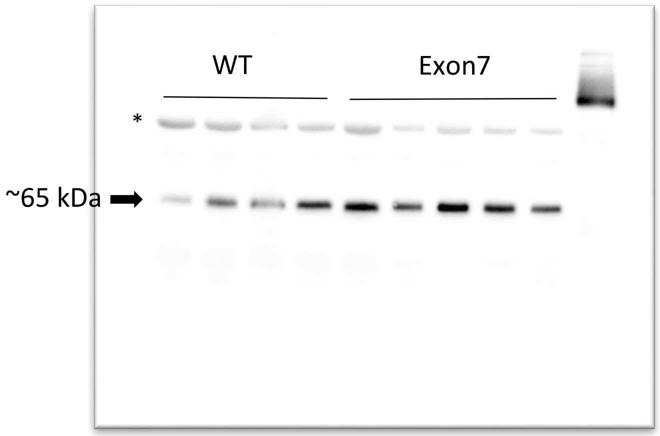

*>150 kDa

SOX4 cells

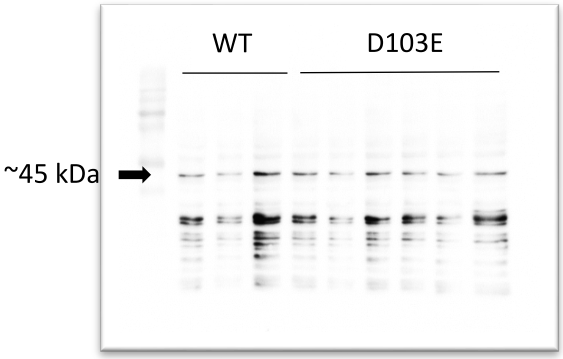

actin

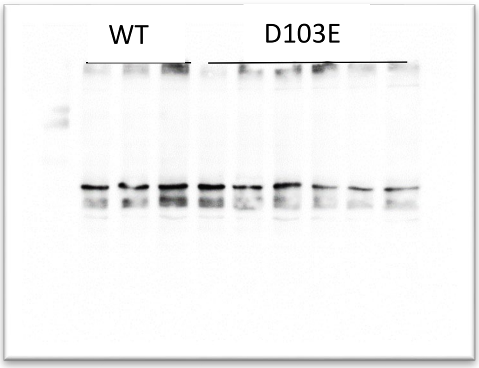

SOX4 cells

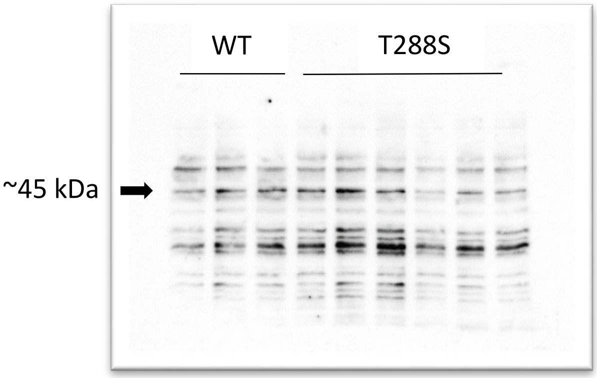

actin

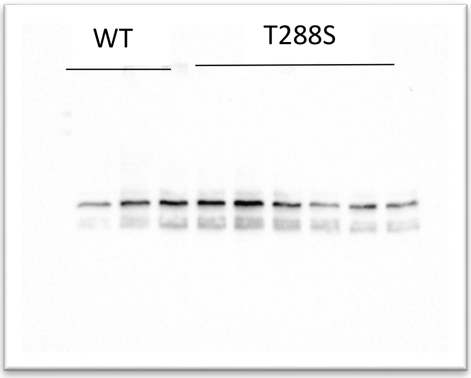

SOX4 cells

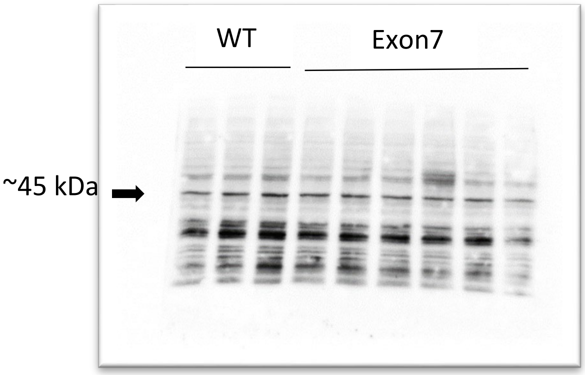

actin

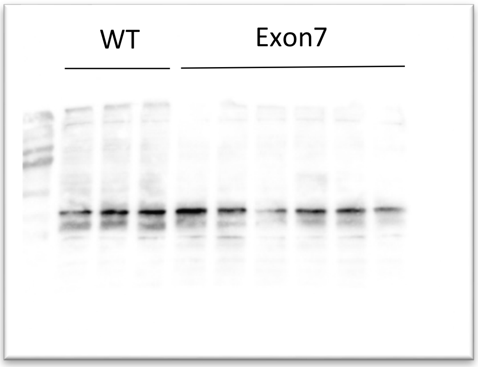

VAMP7 cells

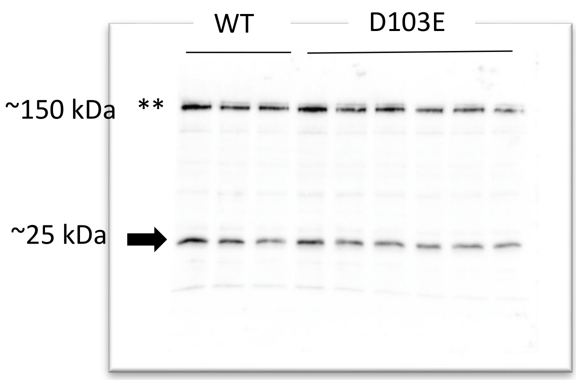

actin

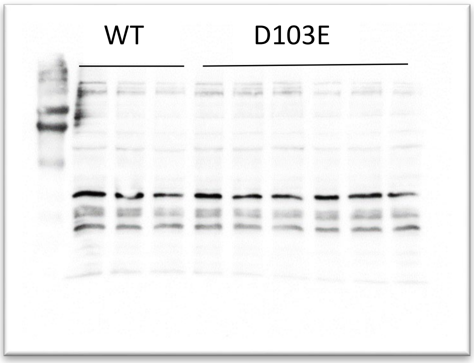

VAMP7 cells

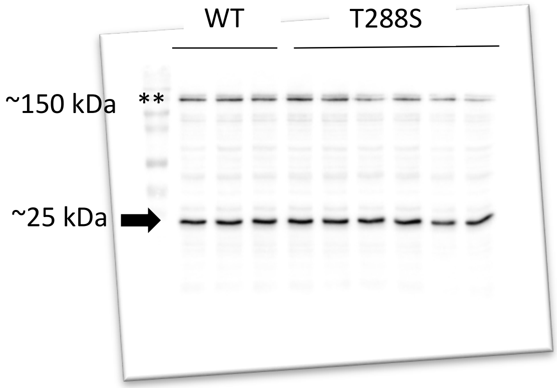

actin

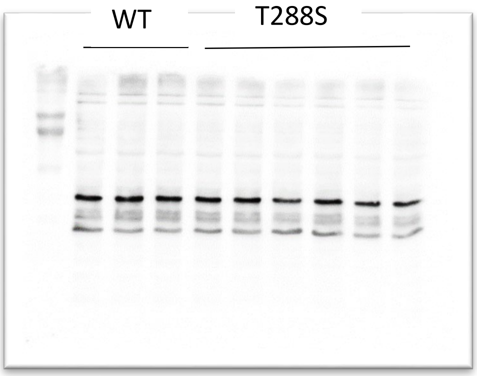

VAMP7 cells

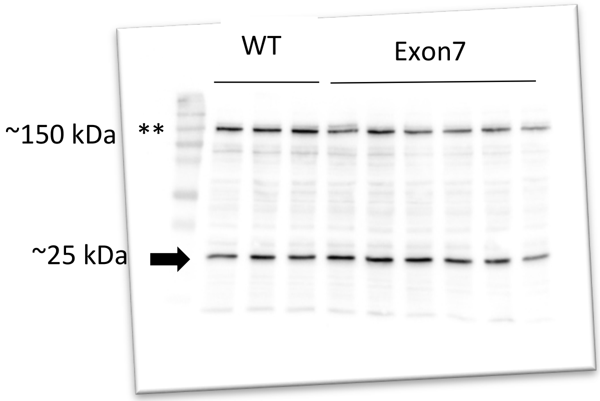

actin

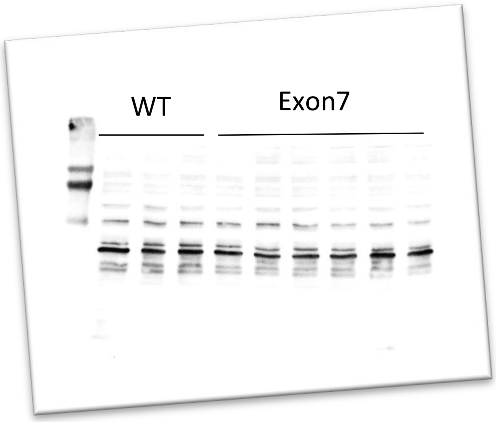

** VAMP7 multimer of unknown function (Siddiqi et al, J Cell Sci, 2006)

PRLR cells

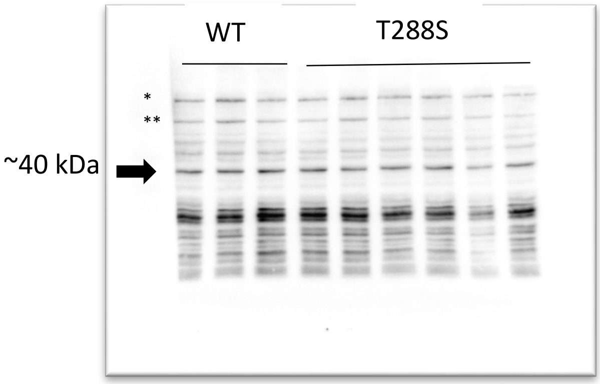

actin

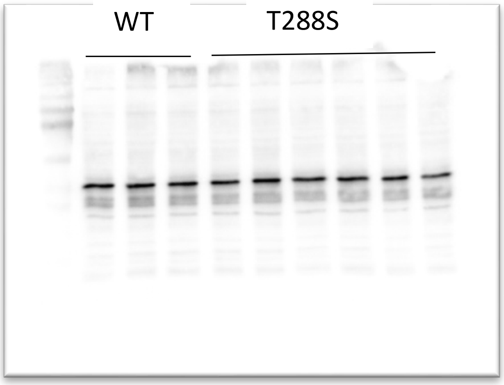

PRLR cells

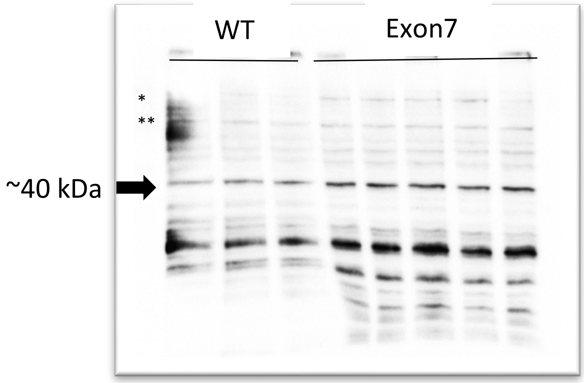

actin

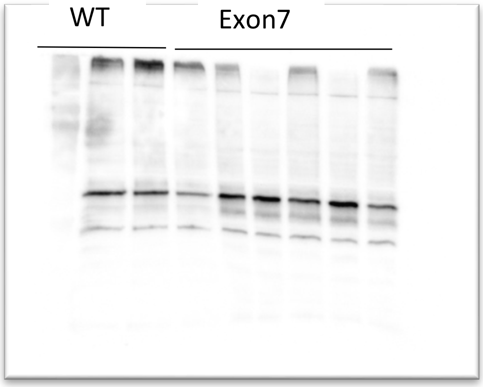

*Long form
**Intermediate form

} Expression pattern similar to short (40 kDa) form, but difficult to detect under these experimental conditions
